# Supplementary material for: Elucidating the genetic architecture of migratory timing in a songbird migrant, the great reed warbler, Acrocephalus arundinaceus
Source: Biol Open. 2025 Nov 7;14(11):bio062039. doi: 10.1242/bio.062039 (PMC12641484; doi:10.1242/bio.062039)
Supplement: Supplementary information [file biolopen-14-062039-s1.pdf]

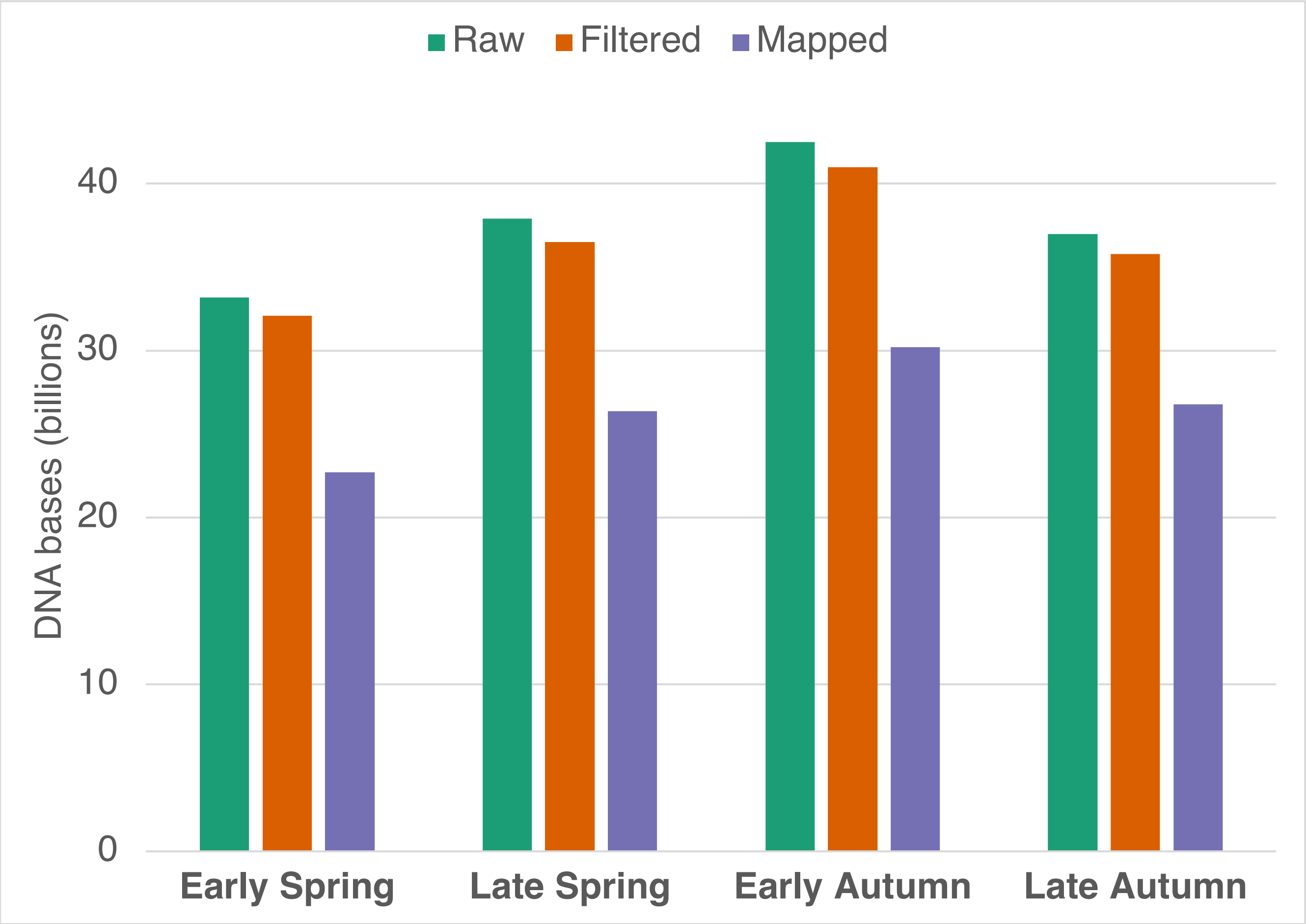

Fig. S1. The number of DNA base pairs at each stage of data processing for each pool.

Biology Open • Supplementary information

Table S1. Individual and migratory data for the great reed warblers utilized in the study.

Pool	Sex	ID	Departure Day	Year
Early Spring	Male	ZA48338	87	2017
Early Spring	Male	ZA24479	91	2016
Early Spring	Male	ZA10292	92	2013
Early Spring	Male	DK13980	92	2015
Early Spring	Male	ZA35998	93	2016
Early Spring	Male	ZA48339	93	2018
Early Spring	Female	ZA24514	92	2013
Early Spring	Female	Z784791	107	2016
Early Spring	Female	ZA18222	107	2016
Late Spring	Male	ZA27389	105	2013
Late Spring	Male	ZA43582	110	2016
Late Spring	Male	ZA43507	114	2015
Late Spring	Male	ZA20410	119	2016
Late Spring	Male	ZA69517	121	2018
Late Spring	Male	ZA59713	129	2017
Late Spring	Female	ZA43567	113	2016
Late Spring	Female	ZA21775	119	2013
Late Spring	Female	ZA43571	133	2016
Early Autumn	Female	ZA45656	182	2017
Early Autumn	Female	ZA20416	193	2015
Early Autumn	Female	ZA22089	203	2014
Early Autumn	Male	ZA76621	184	2017
Early Autumn	Male	ZA24402	193	2014
Early Autumn	Male	ZA48338	197	2016
Early Autumn	Male	DK13980	198	2014
Early Autumn	Male	ZA43523	199	2014
Early Autumn	Male	ZA44461	202	2015
Late Autumn	Female	ZA24514	214	2012
Late Autumn	Female	ZA43571	214	2015
Late Autumn	Female	ZA45738	220	2014
Late Autumn	Female	ZA35959	224	2014
Late Autumn	Male	ZA76610	203	2017
Late Autumn	Male	ZA22062	209	2015
Late Autumn	Male	ZA60716	216	2016
Late Autumn	Male	ZA27389	218	2014
Late Autumn	Male	ZA35003	222	2014

Biology Open • Supplementary information

Table S2. List of 93 candidate genes under selection and the corresponding gene ontology details.

Available for download at
https://journals.biologists.com/bio/article-lookup/doi/10.1242/bio.062039#supplementary-data
